# Bacteriophages and Biofilms

**DOI:** 10.3390/antibiotics3030270

**Published:** 2014-06-25

**Authors:** David R. Harper, Helena M. R. T. Parracho, James Walker, Richard Sharp, Gavin Hughes, Maria Werthén, Susan Lehman, Sandra Morales

**Affiliations:** 1 AmpliPhi Biosciences, Glen Allen, VA 23060, USA; E-Mails: hparracho@thenativeantigencompany.com (H.M.R.T.P.); sml@ampliphibio.com (S.L.); spm@ampliphibio.com (S.M.); 2 Public Health England, Porton Down, Salisbury SP4 0JG, UK; E-Mails: jimmy.walker@phe.gov.uk (J.W.); richard.sharp@phe.gov.uk (R.S.); 3 Gavin Hughes—The Surgical Materials Testing Laboratory, Bridgend, South Wales CF31 1RQ, UK; E-Mail: gavin@smtl.co.uk; 4 Maria Werthén, Mölnlycke Health Care AB, SE-402 52 Gothenburg, Sweden; E-Mail: maria.werthen@molnlycke.com; 5 Department of Biomaterial Science, University of Gothenburg, SE-405 30 Gothenburg, Sweden

**Keywords:** bacteriophage, biofilm, antibiotic resistance, phage therapy, depolymerase, persister cells

## Abstract

Biofilms are an extremely common adaptation, allowing bacteria to colonize hostile environments. They present unique problems for antibiotics and biocides, both due to the nature of the extracellular matrix and to the presence within the biofilm of metabolically inactive persister cells. Such chemicals can be highly effective against planktonic bacterial cells, while being essentially ineffective against biofilms. By contrast, bacteriophages seem to have a greater ability to target this common form of bacterial growth. The high numbers of bacteria present within biofilms actually facilitate the action of bacteriophages by allowing rapid and efficient infection of the host and consequent amplification of the bacteriophage. Bacteriophages also have a number of properties that make biofilms susceptible to their action. They are known to produce (or to be able to induce) enzymes that degrade the extracellular matrix. They are also able to infect persister cells, remaining dormant within them, but re-activating when they become metabolically active. Some cultured biofilms also seem better able to support the replication of bacteriophages than comparable planktonic systems. It is perhaps unsurprising that bacteriophages, as the natural predators of bacteria, have the ability to target this common form of bacterial life.

## 1. Introduction: Biofilms

Biofilms are aggregations of cells, which may be eukaryotic or prokaryotic in nature, surrounded by a matrix of extracellular polymeric substance (EPS) produced, at least in part, by the cells within the biofilm [1]. This EPS consists of long chain sugars, DNA and other biological macromolecules [2], the precise nature of which can be highly variable.

Bacteria within a biofilm can show high levels of resistance to agents, such as biocides and antibiotics. The level of such agents that is needed to produce antibacterial effects can be over 1000 times greater than the level required for free-living (planktonic) bacteria [3]. Suggestions that the biofilm matrix forms an impermeable barrier to all such agents are now thought to be an oversimplification. Some agents may be at least partially blocked, for example the sequestration of tobramycin reported by Mah *et al.* [4]. In contrast, others can readily penetrate the biofilm matrix, as reported for ofloxacin and peracetic acid by Spoering and Lewis [5].

The presence of different conditions (including gaseous, as well as nutrient stratifications) leads to different cell states and, thus, to the existence of distinct zones within the biofilm. Quorum sensing, where cells communicate by releasing small chemical molecules as a signaling process to other cells, results in the biofilm acting in many ways as a community rather than simply a cluster of independent cells [2].

It is now thought that much of the apparent resistance or regrowth of bacteria within the biofilm arises from the presence within the matrix of metabolically inactive “persister” cells. The slow growth and severely limited metabolic activity of these persister bacteria may prevent the action of many antibiotics. Such cells can reactivate after such stress, leading to the regrowth of the biofilm after treatment [6].

Given the ubiquity and advantages of biofilm growth, it is perhaps not unexpected that biofilms appear to be associated with the majority of bacterial infections [2]. Biofilms are thought to underlie much of the reported resistance to antibiotics in clinical use. Such resistance can be apparent *in vivo*, even when the cells themselves, outside the biofilm, such as in the suspended or planktonic state, are susceptible to such agents and, thus, show up as sensitive in assays that do not permit biofilm formation.

**Figure 1:**
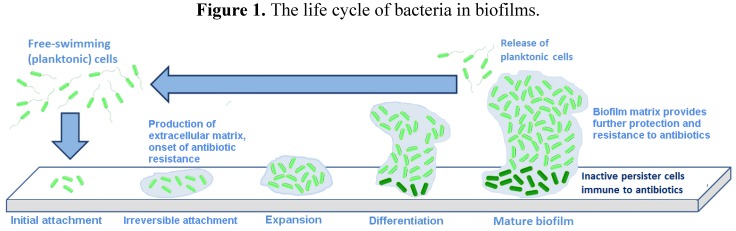
The life cycle of bacteria in biofilms.

An outline of the life cycle of bacterial biofilms is presented in Figure 1, exemplified by the motile bacterium, *Pseudomonas aeruginosa*, which has been used in many studies in this area [7,8]. It should be noted that non-motile bacteria, such as *Staphylococcus aureus*, also form biofilms.

## 2. Bacteriophage and Their Effects on Biofilms

Bacteriophages (often known simply as phages) are naturally occurring viruses that infect bacteria. As such, they are unaffected by antibiotic resistance and (unlike many antibiotics) are able to target bacteria within biofilms [1]. They can either coexist with their host by inserting themselves into the bacterial genome (lysogenic bacteriophages) or destroy them (lytic bacteriophages; the type most suited to therapeutic use). Lytic bacteriophages replicate inside their hosts, then release many new bacteriophages able to infect more bacteria.

It has often been assumed that biofilms confer resistance to bacteriophages, due to the impermeability of the biofilm matrix. However, although they are far larger than chemical antibiotics, bacteriophages are still far smaller than their bacterial hosts, and many bacteriophages can and do infect bacteria within biofilms.

**Figure 2:**
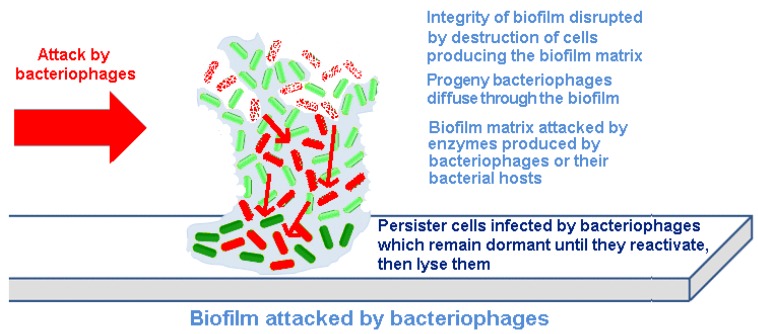
Destruction of a biofilm by bacteriophages.

Bacteriophages act differently on bacteria contained within biofilms than do chemical antibiotics or biocides. Indeed, one can make the argument that phage have co-evolved with bacterial biofilms, and thus, their infection of adherent bacterial populations would be expected. There are at least four mechanisms underlying this difference (Figure 2).

1. Bacteriophages replicate within their host cells, resulting in localized increases in bacteriophage numbers (amplification). This releases increasing numbers of infectious progeny bacteriophages into the biofilm. By spreading through the biofilm, eliminating the bacteria producing the EPS material, bacteriophages can progressively remove the biofilm and reduce the potential for regeneration.
2. Bacteriophages can carry or express depolymerizing enzymes that degrade the EPS.
3. Bacteriophages can induce depolymerizing enzymes that degrade the EPS from within the host genome.
4. Persister cells can be infected by bacteriophages; although bacteriophage cannot replicate within and destroy inactive cells, they can remain within these bacteria until they reactivate and then commence a productive infection, which then destroys the cells.

Bacteriophages can kill their host cells without replicating when they greatly outnumber their bacterial targets, a process called “lysis from without” [9], but such numbers are rarely achieved outside the laboratory. Use of lower numbers of bacteriophages kills the host cells by replication within them followed by lysis during the release of progeny bacteriophages, which then go on to destroy yet more bacteria. Such an infection results in the localized amplification of bacteriophages and can result in self-sustaining infections, with localized spread and ongoing amplification, where the host bacteria are present at levels high enough to support this. However, where insufficient host bacteria are present, this may interrupt the infectious cycle. Given that biofilms are both very common and the location of high levels of bacteria, it is likely that the effective targeting of bacteria within biofilms by bacteriophages represents an evolutionary adaptation to use this rich resource. The mechanisms that they use for this are likely to be based on their need to deal with bacterial capsular polysaccharides during the normal course of infection. This form of infectious spread within a biofilm, with lysis of bacteria as the key element, has been referred to as “active penetration” [10].

It is known that many bacteriophage genomes contain genes for enzymes capable of breaking down elements of the biofilm matrix [1,11,12]. In many cases, these are soluble enzymes that target the host bacterial cell wall during the release from the host cell, but these also have the potential to degrade the biofilm EPS when released from lysing host cells. These cells also release DNA, which can contribute to the biofilm matrix, but can also release DNase enzymes, which are present in the host cells as part of normal replication.

In addition to this, many bacteriophages, such as the T4 and HK620 bacteriophages of *Escherichia* *coli* have enzymes present on the tail of the virus particle, where they aid the penetration of the bacterial cell wall. While they could theoretically play a role in degrading the biofilm matrix, they are often masked until the tail reconfigures during infection and, thus, have a very localized action [11,12]. The requirements for these proteins are precise, since they have to fit into and function within the virus structure. The presence of such enzymes within the tail seems to be a common feature of bacteriophage infection, noted by Yan *et al.* [12] as the “general model of tailed bacteriophage infection”. In this model, components of the bacteriophage tail recognize and digest the capsular polysaccharide, thus allowing the tail to contact the cell membranes through which it then injects the bacterial genome.

Yan *et al*. [12] further notes that “polysaccharide depolymerase protein is a common constituent of the tail structure of a bacteriophage” and that “many tailspike proteins have endoglycosidase activity, hydrolyzing their polysaccharide receptors”. However, this activity is restricted as noted above.

As well as the carriage of the genes coding for such activity, it has long been known that some bacteriophages can induce the expression of depolymerase enzymes in their host bacteria [13]. However, until genome sequencing became possible, it was very difficult to confirm whether this was encoded by the bacteriophage itself.

**Figure 3:**
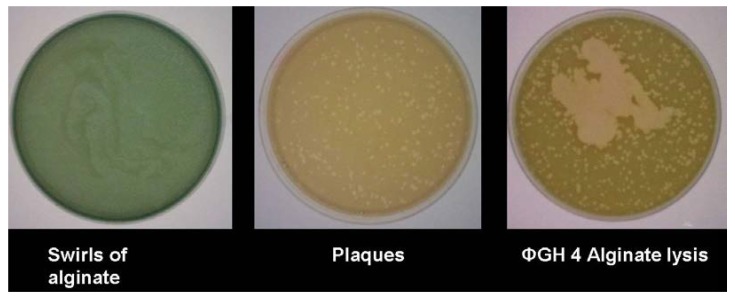
Expression of alginase in bacteriophage-infected *Pseudomonas aeruginosa*.

With bacteriophage GH4, the expression of alginase activity was seen in assays *in vitro* (Figure 3). Despite early suggestions that an alginase was encoded within the bacteriophage genome [14], sequencing showed no such gene, indicating that this alginase is induced from within the host cell. It has been suggested that this could be either a bacteriophage-induced method of rendering the biofilm matrix more porous, thus aiding infection by progeny bacteriophage, or, alternatively, a flight response by infected bacteria, seeking to facilitate movement away from the focus of infection.

The ability to infect persister cells and to remain within them until they reactivate, then to initiate a productive, lytic infection was reported by Pearl *et al.* [15], who stated that “Intriguingly, we found that, whereas persistent bacteria are protected from prophage induction, they are not protected from lytic infection. Quantitative analysis of gene expression reveals that the expression of lytic genes is suppressed in persistent bacteria. However, when persistent bacteria switch to normal growth, the infecting phage resumes the process of gene expression, ultimately causing cell lysis”. This is likely to be a significant element in the bacteriophage-mediated destruction of biofilms.

Supporting this, studies with a static phase culture of *Pseudomonas aeruginosa* embedded in a collagen matrix modeling biofilm wound infection [16] showed no killing of bacteria, even during prolonged incubation (up to five days), until the matrix was disrupted and bacterial growth resumed. Killing by bacteriophages was then readily apparent in the growing cultures [17]. Indeed, such activity can actually be problematical in assessing bacterial numbers after bacteriophage treatment.

## 3. Depolymerases

A large number of enzymes exist that are capable of depolymerizing components of the bacterial biofilm EPS. Among bacteriophages, these include both some of the enzymes produced to release bacteriophages from the host cell (these also include endopeptidases) and tailspike proteins that aid infection, but which are usually restricted to highly localized activity. However, it has been suggested that such proteins, while restricted in activity within the virus particle, can be released from lysing cells in a more generally active form, which can affect the biofilm matrix [18].

It is important to note that different species of bacteria produce different EPS components. Thus, a depolymerase active against the polysaccharides produced by one species may not digest that produced by other bacteria. However, depolymerases are likely to have broader activity than their parent bacteriophages among closely related bacteria, since the complexity (and, hence, the variability) in the EPS is lower than that of the host bacteria. Son *et al.* [19] observed this by comparing the activity of a bacteriophage of *Staphylococcus aureus* with that of the depolymerase that it produced. However, neither would affect bacteria other than *Staphylococci*, suggesting that multiple depolymerases would be required for targeting mixed biofilms.

Where an active depolymerase is produced and released, distinctive “haloes” may be seen around the bacteriophage plaques formed on bacterial lawns, where the bacterial polysaccharide has been damaged or destroyed. Gutiérrez *et al.* [20] used this approach to detect such activity in two bacteriophages infecting *Staphylococcus epidermidis*, both of which were then confirmed by sequencing to contain genes for pectin lyases, while Glonti *et al* [21] identified haloes in cultures of a bacteriophage infecting *Pseudomonas aeruginosa* and purified a depolymerase protein from the bacteriophage using electrophoresis.

Bacteriophage polysaccharide depolymerases have been classified by Yan [12] as endorhamnosidases, alginate lyases, endosialidases and hyaluronidases (glycoside hydrolases). All are known to be carried by multiple bacteriophages. Other relevant enzymes also exist. For example, Broudy *et al.* [22] identified a secreted DNase enzyme produced by a bacteriophage infecting *Streptococcus pyogenes*.

## 4. Bacteriophage Growth in Biofilms

**Figure 4:**
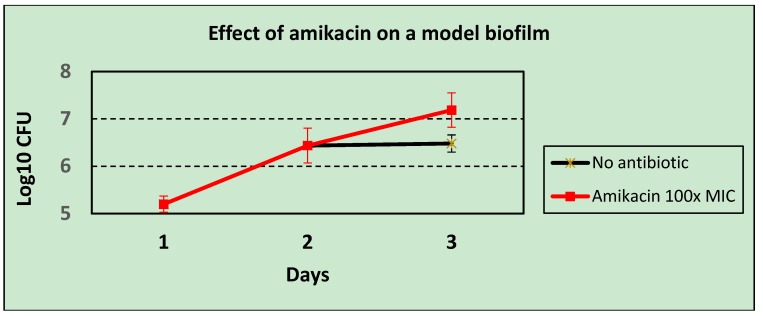
The effect of high dose antibiotic (Amikacin at 100× MIC) on an early-stage biofilm of *Pseudomonas aeruginosa*.

Experimental data indicates that bacteriophages grow well in *Pseudomonas aeruginosa* biofilms [23], at least in the early stages of biofilm growth. Using two-day-old biofilms [3], it was found that of 17 strains of *Pseudomonas aeruginosa* that were insensitive to a test set of bacteriophages in a plaque assay (thus, using planktonic bacterial hosts), 8/17 strains supported the growth of the same bacteriophages in the biofilm model. These early-stage biofilms are, however, able to block the activity of antibiotics, with Amikacin at 100× the MIC actually enhancing bacterial growth in this system (Figure 4), even though the bacterium was shown to be sensitive at the MIC by disc testing when growing planktonically. This accords with the findings of Gupta *et al.* [8], who also found that the onset of antibiotic resistance occurred in the early stages of biofilm development. Thus, bacteriophages were shown to be able to kill bacteria in situations where conventional antibiotics cannot do so.

In early studies that helped to demonstrate the potential of bacteriophages for biofilm control, Hanlon *et al.* [24] found that *Pseudomonas aeruginosa* bacteriophages could destroy bacteria in a mature (20-day-old) biofilm and (perhaps surprisingly, given their size) could diffuse through even the thickest (12%) alginate gel studied, although diffusion was slower than through thinner alginate gels. Hanlon also observed that the bacteriophages studied could degrade the alginate polymer directly, apparently via a bacteriophage-carried enzymatic activity, although this was not identified. Whatever the activity, it was clearly different from the highly-restricted tailspike proteins.

Sillankorva *et al.* [1] used bacteriophages of both *Pseudomonas fluorescens* and *Staphylococcus* *lentus* and demonstrated the effective reduction of single species and mixed biofilms with these agents. Both bacteriophages were fully sequenced, and it was demonstrated that neither coded for a polysaccharide depolymerase (although the *Pseudomonas fluorescens* bacteriophage encoded an endopeptidase). Similarly, Doolittle *et al.* [25] showed that *Escherichia coli* bacteriophage T4 spread efficiently through a biofilm, although it does not code for any polysaccharide depolymerases other than a very restricted tailspike protein, which is only broken out of the bacteriophage tail during the penetration of the host cell. However, Doolittle *et al.* [25] also worked with the E79 bacteriophage of *Pseudomonas aeruginosa* and showed that this was less effective than T4 at penetrating biofilms.

Although it is clear that naturally occurring bacteriophages can penetrate biofilms even when they do not produce polysaccharide depolymerases (or when these are of a very restricted function), not all studies have shown efficient infection within biofilms and some workers continue to believe that EPS-degrading enzymes are necessary for biofilm applications [18].

Tait *et al.* [26] reported that a mixture of three bacteriophages could completely eliminate a single species biofilm, but that this was less effective when other, insensitive bacterial species were present. Kay *et al.* [27] also showed that mixed biofilms could ablate the efficacy of bacteriophages. Despite this, Sillankorva *et al.* [1] showed that efficiency in model biofilms could be high, even with a bacteriophage targeting a single bacterial species, stating that “phages can be adopted as a method to kill a specific bacterium even when its host resides in mixed consortium”. Sillankorva *et al*. [1] also showed that mature (seven-day-old) biofilms could be targeted effectively using bacteriophages.

Thus, it is clear that natural bacteriophages can and often do express enzymes capable of disrupting biofilms, but that these do not seem to be essential for infectivity in this situation. The potential for the induction of such enzymes from the host genome is, of course, far harder to identify.

## 5. Enhancing the Activity of Bacteriophages

One concern over the commercial development of bacteriophages as control agents has historically been the opinion that it was not possible to patent natural bacteriophages. This is incorrect as demonstrated by patents awarded to multiple groups protecting such bacteriophages, based, as for all patents, on novelty and “surprising” activity [14,28,29]. However, a number of companies have attempted to use engineered bacteriophages, which has the effect of enhancing the novelty inherent in their intellectual property position.

One approach to this is the addition of virulence-enhancing factors to the bacteriophage genome. This was the approach taken by Lu and Collins [30], who inserted a gene for Dispersin B (a glycoside hydrolase known to have biofilm-dispersing activity) into *Escherichia coli* bacteriophage T7. This was then expressed at high levels during infection, being released into the extracellular matrix from lysed cells. Lu and Collins [30] found that this substantially improved biofilm removal compared to the parent T7 (which was itself a recombinant, containing a gene from bacteriophage T3 to permit replication in this system). T7 naturally produces an endopeptidase, but does not produce a polysaccharide depolymerase. This appears to have represented an experimental system selected to highlight the effect of the expressed enzyme, since Lu and Collins [30] noted that *“*E. coli, which produces the K1 polysaccharide capsule, is normally resistant to infection by T7 phage” but that this contrasts with Escherichia coli bacteriophage T4, “which can infect and replicate within Escherichia coli biofilms and disrupt biofilm morphology by killing bacterial cells”. This approach was then entered into commercial development, targeted at use in biofilm removal in industrial processes.

The value of this type of approach, with the additional regulation inherent in the use of genetically modified organisms (GMOs), has of course to be considered alongside the demonstrated ability of at least some natural bacteriophages to target biofilms effectively. Clearly, generating novel recombinant bacteriophages in this way does enhance patentability, but this has to be considered alongside the potential for developing natural bacteriophages for this purpose.

It should be noted that Lu and Collins [30] used a soluble enzyme, released from the infected cell by lysis. This is relatively straightforward compared to engineering expression within the virus particle, where there are far more constraints on activity unless more permissive methods, such as phage display, are used.

The alternative approach of co-administering bacteriophages with biofilm dispersing agents, such as alginate lyases or even DNase enzymes (such as the commercial product Pulmozyme), has also been suggested [14]. However, this would not permit such agents to be present for succeeding generations of bacteriophage, unlike those that are expressed from within the bacteriophage genome.

## 6. Biotic and Abiotic Systems

**Figure 5:**
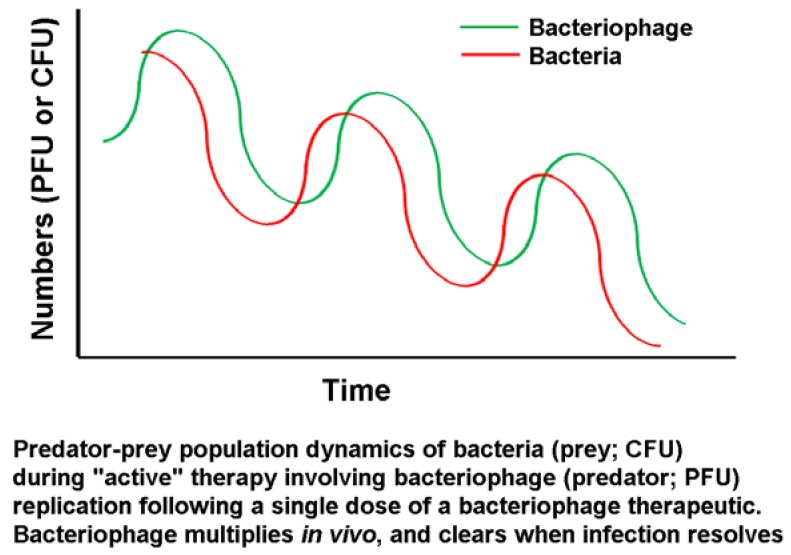
Predator-prey cycles as applied to bacteriophages [30].

Bacteriophages are replicating biological control agents and are thus inherently different from chemical agents, as noted above. Given the replicating nature of the agent, when the levels of host cells fall below critical (low) levels, bacteriophage amplification is no longer sustainable. However, the work of Marza *et al.* [31] showed that even a picogram-range dose (400 PFU) of a single bacteriophage type is capable of resolving a chronic infection *in vivo*. This single case study showed cycles of improvement and deterioration in the clinical condition, albeit with an overall positive trend, leading to successful resolution after several months. This aligns with the expectations of predator-prey cycles in such a system, as illustrated by Parracho *et al.* [32] (Figure 5).

In an *in vivo* system, other factors are acting alongside bacteriophages, which act to eliminate an infection. As well as the effects of the immune system, infected material may be cleared by biological effectors (for example, by ciliary action in the lung or ear), mechanically (by wound debridement or physical removal from the upper regions of the lung), chemically (by the disinfection of burns) or even by inherent properties of the infection itself (for example, by the shutdown of pathogenic effectors via quorum sensing when bacterial numbers drop below critical levels). Bacteriophages are a form of biological control [33] and, as such, act in concert with other effectors. This is illustrated by the observation that the efficacy of bacteriophage therapy *in vivo* is reduced when the host animal is immunosuppressed [34].

When bacteriophages are used therapeutically *in vivo*, they have been shown to be able to clear infections, even where these are characterized by biofilm formation, for example in work with chronic infections of the ear in both animal and human systems [31,35,36]. Successful clearance is likely to arise from a combination of direct antibacterial effects and other effectors, as noted above.

In an abiotic system, such as industrial fouling by biofilms, there is a lack of such additional factors, and this may be why enhancing the activity of bacteriophages has attained greater emphasis in such systems. Many of the systems used to study the effects of bacteriophages on biofilms are simple, *in vitro* systems where such effects might be anticipated [26]. However, Sillankorva *et al.* [1] has shown the efficient clearing of both single and multispecies biofilms cultured on steel plates by bacteriophages, so this is by no means an absolute.

## 7. Combination with Other Agents

Verma [37] noted that lytic bacteriophages could make mature biofilms more amenable to antibiotic treatment, which aligns with findings from the few clinical trials of bacteriophage efficacy reported to date [28,38]. Based on this, the combined or sequential use of antibiotics and bacteriophages has been identified as having potential for therapeutic applications. Supporting this, Yilmaz *et al.* [39] showed increased effects when bacteriophages and antibiotics were used in combination, including clearance of *Staphylococcus aureus* biofilms. Other combinations are also possible. Sharp *et al.* [14] proposed the use of a polysaccharide lyase and of DNase enzymes to degrade the biofilm matrix, to be administered alongside bacteriophages. This is also discussed by Abedon [40], although differential diffusion of bacteriophages and co-administered enzymes is noted as an issue. Physical cleaning of wounds can also be combined with bacteriophage use. Seth *et al.* [41] found using a rabbit ear model that, while neither wound debridement nor phage treatment alone produced an effect in this system, a combination of both was effective.

Similarly, for industrial biofilms, Sillankorva *et al*. [1] noted that “Although phages can decrease bacterial populations present in biofilms, these biological agents alone are most likely not efficient enough to be applied to control industrial biofilms. Commonly used cleaning procedures remove not only microorganisms, but also all undesirable materials (e.g., foreign bodies, cleaning chemicals, soil, *etc.*), and phages are not capable of this task. However, phages could have a similar function to nowadays used biocides and sanitizers, and be used after the major cleaning processes, to kill specific bacterium on the remainder biofilms.” Similarly, Ganegama Arachchi *et al*. [42] showed efficient clearance of *Listeria monocytogenes* biofilms from steel surfaces by a mixture of three bacteriophages, even when these were contaminated with fish protein. However, Ganegama Arachchi *et al.* [42] also found that this was accelerated by mechanical clearing, stating that “Phages were more effective on biofilm cells dislodged from the surface compared with undisturbed biofilm cells. Therefore, for short-term phage treatments of biofilm it should be considered that some disruption of the biofilm cells from the surface prior to phage application will be required”. As with biological systems, other combinations are possible. Liao *et al*. [43] observed synergistic effects on silicone catheter segments in the prevention of biofilm formation when bacteriophages were combined with commensal bacteria, while Zhang and Hu [44] observed increased effects on filters when combining bacteriophages with a biocide (chlorine).

## 8. Engineered and Natural Bacteriophages

As with any biological system, it is possible to optimize bacteriophages for specific functions by either GM approaches [30] or, more recently, by the use of synthetic biology. In doing so, one has to be aware that there are likely to be additional regulatory requirements related to such products [40]. For example, the French company, Pherecydes Pharma, was established to explore the use of such approaches [45]. However, in 2011, they reported that the regulatory complexities surrounding the use of GM agents had refocused their activities on natural bacteriophages [46]. They are currently a main participant in the multicenter EU-sponsored PhagoBurn clinical trial, using natural (non-recombinant) bacteriophages [47].

Bacteriophages are thought to be the most numerous form of life, with around 10^31^ bacteriophages present in the global biosphere [48]. Of these, only a few thousand have been sequenced. Rohwer [49] estimated that “less than 0.0002% of the global phage metagenome has been sampled”. As a result, most bacteriophage genes are unknown. Even where the genomes have been sequenced, many of the genes are ORFans; that is, unique genes with no known homologues in other organisms [50]. This results from the highly diverse nature of bacteriophages and means that it is extremely difficult to map the functional elements of bacteriophage replication. To support attempts to enhance or design bacteriophages, there needs to be a far greater understanding of the basic genetics of the systems under study, accompanied by expanding knowledge of just what is present in existing ecosystems. There are signs that technology is beginning to deliver such an understanding [51], but this remains at an early stage.

As a result of the massive and often readily available diversity of bacteriophages, sampling from the environment can be used to identify and isolate naturally evolved bacteriophages for almost any target or application, including biofilms. It is further possible to optimize these bacteriophages for their intended applications either in the initial selection or by serial passage or other standard techniques [52]. This approach can also be used to identify multiple agents, allowing for the possibility of substitution into an approved product [53], which could be more difficult to attain with highly developed GM or other products based around a small number of agents.

## 9. Conclusions

Bacteriophages possess unique properties and show considerable promise in the control of biofilms. However, such applications are still evolving, and large-scale uses are still under development. Thus, the identification of the most effective approaches has to be, at present, speculative in nature. In time and as more results are published, best practices for such uses will, of course, emerge.
